# Spatial Patterns of Intraspecific Genetic Diversity Follow no General Rule Across Climatic and Geographic Gradients

**DOI:** 10.1111/mec.70321

**Published:** 2026-03-24

**Authors:** Matthew O. Moreira, Maria J. Paúl, André V. Liz, Ana C. Carnaval, Bryan C. Carstens, Sílvia B. Carvalho

**Affiliations:** ^1^ CIBIO, Centro de Investigação em Biodiversidade e Recursos Genéticos, InBIO Laboratório Associado, Campus de Vairão Universidade do Porto Vairão Portugal; ^2^ BIOPOLIS Program in Genomics, Biodiversity and Land Planning CIBIO Vairão Portugal; ^3^ Departamento de Biologia, Faculdade de Ciências Universidade do Porto Porto Portugal; ^4^ GEMAP Research Group, Departamento de Xeografía Universidade de Santiago de Compostela Santiago de Compostela Spain; ^5^ CISPAC, Edificio Fontán, Cidade da Cultura Santiago de Compostela Spain; ^6^ Department of Biology, City College of New York and Biology Program at CUNY Graduate Center City University of New York New York City New York USA; ^7^ Biology Ph.D. Program, CUNY Graduate Center New York City New York USA; ^8^ Department of Evolution, Ecology and Organismal Biology The Ohio State University Columbus Ohio USA

**Keywords:** central‐marginal hypothesis, climatic niche, genetic diversity, geographic range

## Abstract

Intraspecific genetic diversity (ISD) underpins key eco‐evolutionary processes, yet its spatial distribution across species' ranges remains poorly understood at broad scales. Combining mitochondrial sequence alignments, species distribution models and comparative analyses, we tested whether populations closer to current niche optima exhibit higher ISD—a prediction derived from the central‐marginal paradigm. With this aim, we investigated how ISD varies in relation to both climatic and geographic centroids using data from 436 herptile species (248 reptiles and 188 amphibians) from six regions across the world. We adopted a meta‐analysis approach based on publicly available data. For species presenting at least five georeferenced DNA sequences from unique locations (~2.5‐km resolution) within their buffered geographic range, we generated spatially explicit ISD surfaces by interpolating gene‐specific data. We then quantified the relationship between ISD and the distance to both climatic and geographic centroids using species‐specific Spearman's *ρ* coefficients. To further explore the drivers of these patterns, we applied a Random Forest framework to predict Spearman's *ρ* as a function of climate, ecology, geography, morphology and demography. Contrary to prevailing assumptions, the strength and direction of ISD–centroid correlations proved highly variable and species‐specific, and the models consistently showed poor predictive performance. These results suggest that no uniform macroecological or evolutionary processes govern ISD patterns across taxa, but rather that ISD reflects lineage‐specific histories, ecological contexts and demographic contingencies. Our findings underscore the challenges of predicting genetic diversity patterns and highlight the relevance of species‐tailored approaches in conservation planning.

## 1 Introduction

Intraspecific genetic diversity (ISD) is a foundational component of biodiversity, alongside species and ecosystem diversity, and it plays a crucial role in determining species' adaptive capacity, evolutionary potential and ecological stability (Kardos et al. 2021; Lande and Shannon 1996; Sgrò et al. 2011). By influencing both population viability and ecosystem resilience, ISD contributes directly to nature's capacity to sustain ecosystem services that underpin human societies, including food security, disease regulation and long‐term environmental stability (Des Roches et al. 2021). Despite its importance, ISD remains underrepresented in conservation policy and spatial planning efforts (Hoban et al. 2023; Laikre 2010; Laikre et al. 2020; Taylor et al. 2017). Although species distributions and species richness have been widely studied, we still understand relatively little about how ISD is organized—whether across the globe at macrogenetic scales (Miraldo et al. 2016) or within species' ranges along geographic and environmental gradients (Sagarin et al. 2006). However, the growing availability of molecular data (e.g., Blanchet et al. 2017), coupled with recent advances in spatial and statistical methodologies, now provides a unique opportunity to investigate the spatial patterns of genetic diversity and the factors that shape them across species.

One influential hypothesis, the central‐marginal hypothesis, provides a spatial framework for understanding how ISD is distributed within species' ranges. It predicts that ISD peaks near the centre of a species' geographic range and declines towards the periphery, driven by the expectation that larger, more stable and better‐connected populations occur in central areas, whereas smaller, and more isolated edge populations experience stronger drift and reduced gene flow (Eckert et al. 2008; Langin et al. 2017; Lira‐Noriega and Manthey 2014). This hypothesis is rooted in the abundant‐centre model, which posits that species are most abundant in central, suitable habitats and decline in density towards marginal habitats (Langin et al. 2017; Sagarin and Gaines 2002). Extending this idea to ecological space, the niche‐marginality hypothesis suggests that genetic diversity should peak near the climatic‐niche centroid, where environmental conditions are optimal, and decline towards marginal conditions which presumably have comparatively reduced demographic performance (Diniz‐Filho et al. 2009; Lira‐Noriega and Manthey 2014). A complementary framework, the rear‐leading edge hypothesis, highlights the influence of past climatic oscillations on present‐day ISD patterns. It predicts higher genetic diversity in populations that persisted in long‐term climatic refugia (rear edge) relative to those that expanded into new areas during postglacial range shifts (leading edge; Carnaval et al. 2009). Such expansions are often accompanied by demographic bottlenecks, allele surfing and the accumulation of deleterious mutations, processes that collectively erode genetic diversity and increase genetic differentiation in newly colonized regions (Excoffier et al. 2009; Gilbert et al. 2018; Peischl et al. 2013). Despite their strong theoretical underpinnings, empirical evidence for these hypotheses remains mixed, with growing evidence that the spatial structure of ISD is highly variable across clades and regions (e.g., Pironon et al. 2017; Singhal et al. 2022).

Understanding the drivers of ISD heterogeneity is essential not only for testing fundamental macroecological and evolutionary hypotheses but also for identifying conservation priorities in a rapidly changing world (Hughes et al. 2008). However, generalizing ISD patterns is complicated by multiple interacting factors. Demographic processes and taxon‐specific traits, including dispersal ability, can shape how genetic diversity is maintained or eroded across ranges (Barrow et al. 2021). Historical contingencies, such as climatic stability and suitability, leave contrasting signatures of diversity across space, while ecological factors—including hypervolume or climatic‐niche breadth—further mediate genetic patterns in ways that are not always captured by broad‐scale models. Spatial coverage and methodological aspects, such as sampling design and marker choice, add additional layers of variability. Additionally, studies often rely on limited geographic or phylogenetic scopes (Singhal et al. 2022), or meta‐analysis (Pironon et al. 2017), reducing the generalizability of their findings. This complexity underscores the importance of evaluating a broad suite of predictors simultaneously—spanning environmental, ecological, spatial, morphological, genetic and sampling dimensions—to identify which factors, if any, systematically explain variation in ISD ~ distance to centroid correlations.

**FIGURE 1:**
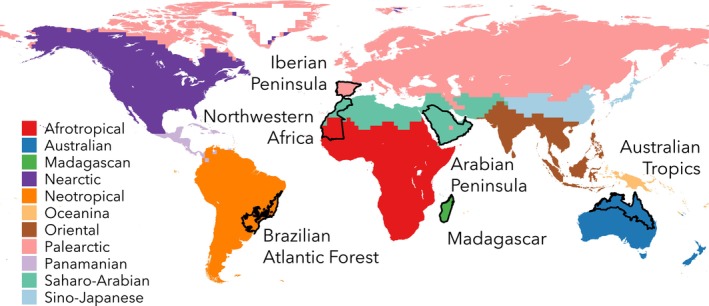
Global map highlighting the six global regions (in black) used in this study: The Iberian Peninsula, Northwestern Africa, the Brazilian Atlantic Forest, the Arabian Peninsula, Madagascar and the Australian Tropics. The background colour palette represents the 11 Zoogeographic Realms defined by Holt et al. (2013), providing biogeographical context for the distribution of the study regions across the globe.

In this study, we aim to overcome these limitations by analysing ISD patterns across 436 species of reptiles and amphibians (hereafter, herptiles) distributed in six ecologically and climatically distinct regions across the globe that have been sampled extensively through studies of ISD: the Iberian Peninsula, the Brazilian Atlantic Forest, Madagascar, the Arabian Peninsula, the Australian Tropics and Northwestern Africa (Figure 1). Herptiles represent an excellent system for investigating large‐scale patterns of genetic diversity. As ectotherms with generally low‐dispersal ability, they are strongly constrained by environmental conditions, making their population dynamics and range structure more closely tied to climate and geography. These taxa are also known to harbour high levels of ISD, providing a solid foundation for cross‐species analyses. At the same time, their strong dependence on climate makes them particularly vulnerable to environmental change (Foden et al. 2013), underscoring the importance of understanding how genetic diversity is distributed in these groups. Using spatially explicit estimates of mitochondrial nucleotide diversity across each species range, we evaluate whether ISD tends to be structured around species' climatic or geographic centroids—used here as proxies for niche optima and central ranges, respectively. Rather than testing these patterns in detail within each species, our goal is to detect broad signals that may emerge when leveraging pooled data across diverse taxa. We quantify the strength and direction of these relationships and implement a machine learning framework to test whether a broad set of ecological, spatial and methodological variables can reliably predict these correlations. Our approach integrates genetic data, species distribution modelling (SDMs) and comparative statistics using pooled data to evaluate whether general rules govern how ISD varies across climatic and geographic gradients.

## 2 Methods

**FIGURE 2:**
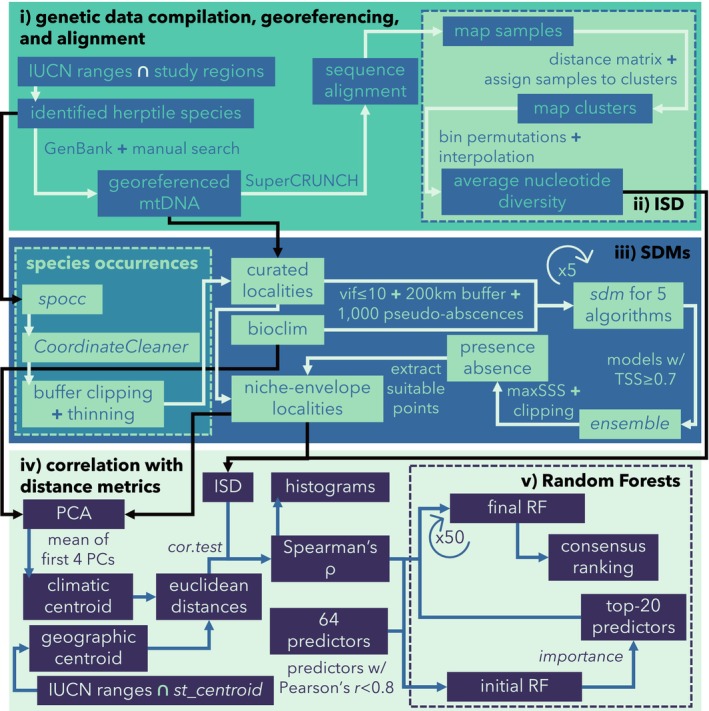
Schematic flowchart outlining the main analytical steps of the study. This pipeline includes genetic data compilation, georeferencing, and alignment, calculation of spatially explicit intraspecific genetic diversity (ISD), estimation of species distribution models (SDMs), correlation analyses between ISD and distance metrics, and the implementation of machine learning to assess potential predictors of Spearman's *ρ*.

The workflow used in this study included five main steps: (i) genetic data compilation, georeferencing and alignment; (ii) inference of spatially explicit ISD surfaces; (iii) inference of species‐specific SDMs; (iv) calculation of correlation coefficients between ISD and both climatic and geographic centroids; and (v) inference of the main predictors of Spearman's rank correlation coefficients (*ρ*) using Random Forests (hereafter, RF; Figure 2). Each of these methodological components is described in detail in the sections below.

### 2.1 Genetic Data Compilation, Georeferencing and Alignment

To identify the species present in our focal regions, we first downloaded all available herpetofauna range maps from the IUCN Red List (IUCN 2024) and overlaid them with the geographic boundaries of our six study areas (Figure 1; Table 1): the Iberian Peninsula, Brazilian Atlantic Forest, Madagascar, Arabian Peninsula, Australian Tropics and Northwestern Africa. We then compiled genetic sequences and associated metadata (e.g., country, locality and coordinate information) for all species occurring in these regions using a custom script built with the *rentrez* R package (Winter 2017) to extract data from GenBank (https://www.ncbi.nlm.nih.gov/genbank/). Our dataset focused on mitochondrial DNA (mtDNA), including both partial gene fragments and complete mtDNA genomes. We retained only those species and genes with at least 10 sequences for sequence alignment, a threshold shown by Barrow et al. (2021) to minimize bias in nucleotide diversity estimates arising from insufficient genetic sampling. Marine and invasive species were excluded from our dataset to avoid the inclusion of lineages likely influenced by non‐comparable ecological or evolutionary dynamics. To enhance spatial coverage, we georeferenced sequences lacking coordinate data on GenBank by searching for geographic details (coordinates and/or locality descriptions) in associated publications when available, manually curated by the authors following standardized procedures and regional expertise. For sequences with locality data but no coordinates, we used the Google Maps application‐platform interface (API) in combination with the *ggmap* R package (Kahle and Wickham 2013) to obtain coordinates based on locality descriptions, filtered by country. Notwithstanding, the majority of sampling localities (i.e., grid cells) were derived directly from coordinates reported in GenBank, reported by regional experts or in associated publications (~88% for reptiles and ~75% for amphibians). Species‐genes combinations with fewer than five georeferenced sequences from unique sampling localities were excluded from further analyses, as interpolation methods do not work below this threshold.

**TABLE 1:** Total number of species per region and number of species included in the study per region for reptiles (*n* = 248 species) and amphibians (*n* = 188 species).

Region	Reptiles		Amphibians	
	Total	This study (%)	Total	This study (%)
Iberian Peninsula	47	32 (68.9)	35	24 (68.6)
Brazilian Atlantic Forest	247	26 (10.5)	525	81 (15.4)
Madagascar	387	45 (11.6)	372	43 (11.6)
Arabian Peninsula	189	63 (33.3)	11	4 (36.4)
Australian Tropics	395	46 (11.6)	148	28 (18.9)
Northwestern Africa	148	59 (39.9)	28	13 (46.4)

*Note:* Values in parentheses indicate the proportion of the total number of species per region represented in this study.

Sequence alignment was carried out for each gene on a species‐by‐species basis using SuperCRUNCH, a Python‐based toolkit designed to assemble large phylogenetic datasets from GenBank and unpublished sequence data (Portik and Wiens 2020). The pipeline included initial parsing of raw sequences, filtering and selecting sequences with a minimum length of 250 base pairs and performing multiple sequence alignments using MAFFT with the accurate adjust setting. Partial sequences were retained provided they overlapped sufficiently to allow reliable alignment. Post‐alignment processing steps included re‐labelling sequence headers for compatibility with downstream analyses and edge trimming to remove gap‐rich regions from the alignments, thereby excluding poorly aligned regions and minimizing the influence of missing data in the final alignments.

### 2.2 Inference of Spatially Explicit ISD Surfaces

We estimated spatially explicit ISD surfaces across the geographic range of each species (Figure S1). To address potential biases caused by uneven sampling density, we applied hierarchical clustering using the unweighted pair group method with arithmetic mean (WPGMA; Sneath and Sokal 1973). The clustering threshold was defined by the first quartile of the distribution of pairwise distances between sampling locations. Based on the resulting clusters, we constructed a set of combination bins equal to the number of locations in the most densely sampled cluster. For each bin, one location was selected per cluster, ensuring that all sampling points were included in at least one bin. Locations in the largest cluster were used only once, while those in smaller clusters could appear in multiple bins. Within each bin, we calculated nucleotide diversity at each location using the ‘nuc.div’ function implemented in the *pegas* R package (Paradis 2010). Nucleotide diversity at individual locations was calculated using a minimum of two sequences, consistent with its pairwise definition. To ensure this minimum, we defined a neighbourhood circle around each sampling location, with the neighbourhood radius set to the maximum nearest‐neighbour distance across individual sequences, thus guaranteeing the inclusion of at least two sequences. Within each defined neighbourhood, we identified unique haplotypes and computed all pairwise comparisons between them. For locations with more than two sequences, all pairwise comparisons were retained, and the final nucleotide diversity value was obtained as the average of these pairwise differences.

To spatially interpolate nucleotide diversity across each species' distribution range, we fitted thin plate spline models using the *fields* R package (Nychka et al. 2021). This allowed us to generate predictive ISD surfaces and estimate standard errors across the range. The entire process—from binning to interpolation—was repeated for each bin, and we then averaged the predictive nucleotide diversity and standard error across all bins for each species. All mtDNA genes for which sufficient data were available were retained in the dataset; however, only the single most representative gene per species (i.e., largest number of georeferenced samples) was used for ISD estimation in the main analysis. For species represented by more than one mtDNA gene, we additionally implemented a multi‐locus approach to calculate average genetic diversity across loci as a complementary sensitivity analysis (see Appendix S1), to assess whether integrating information across multiple genes altered the inferred spatial patterns of ISD.

### 2.3 Inference of Species‐Specific SDMs

For each species, we inferred an ensemble of SDMs. We retrieved occurrence records using the ‘occ’ function from the *spocc* R package (Owens et al. 2024), with the species name plus synonyms (Datasets S1 and S2), and complemented these with the georeferenced genetic data compiled earlier. Taxonomic synonyms were identified by querying the Integrated Taxonomic Information System (ITIS) using the *taxise* R package (Chamberlain and Szocs 2013). This search included records mainly from the Global Biodiversity Information Facility (GBIF; Moreira et al. 2026), as well as VertNet and Integrated Digitized Biocollections (iDigBio). We cleaned the raw species occurrence data using the ‘clean_coordinates’ function from the *CoordinateCleaner* R package (Zizka et al. 2019). To ensure data quality, we removed occurrences falling largely outside the species' known distributions based on IUCN range maps. We applied a variable buffer around IUCN polygons that scaled with the square root of the species' range area—ranging from a 10‐km buffer for very small ranges (≤ 100 km^2^) to a maximum of 100 km for the largest ranges (≥ 8101 km^2^), increasing in 10 km increments. Next, we performed spatial thinning by removing duplicate records occupying the same grid cell at a resolution of ~2.5 km. This yielded a final dataset of filtered and standardized occurrence records for each species, referred to hereafter as curated localities.

To derive the climatic centroid for each species, we developed an ensemble of species‐specific SDMs using the ‘ensemble’ function from the *sdm* R package (Naimi and Araújo 2016). The models were calibrated using the curated localities in combination with bioclimatic variables sourced from CHELSA v2.1 (Karger et al. 2017, 2018) at ~1 km resolution (30 arc‐s). The climatic predictors included seven widely used variables capturing annual extremes for temperature (Bio5: mean daily maximum air temperature of the warmest month; Bio6: mean daily minimum air temperature of the coldest month) and precipitation (Bio16: mean monthly precipitation amount of the wettest quarter; Bio17: mean monthly precipitation amount of the driest quarter), both annual temperature and precipitation averages (Bio1: mean annual air temperature; Bio12: annual precipitation amount) and isothermality (Bio3). These variables are known to influence species' distributions due to their physiological and ecological relevance (Araújo et al. 2006; Mi et al. 2023). To minimize multicollinearity among predictors, we used the ‘vifstep’ function from the *usdm* R package (Naimi et al. 2014) to remove any variable with a variance inflation factor (VIF) exceeding 10. This procedure was applied independently for each species.

We defined the calibration area for each SDM as a 200‐km buffer surrounding the curated localities, constrained within the relevant Wallace's Zoogeographic Regions where each species occurs (Holt et al. 2013), to ensure ecologically meaningful predictions (Merow et al. 2013; Mi et al. 2023; VanDerWal et al. 2009). Within this area, pseudo‐absence points were randomly sampled to match the number of occurrence records per species, with a minimum of 1000 pseudo‐absences (Barbet‐Massin et al. 2012; Mi et al. 2023), using the ‘sdmData’ function from the *sdm* R package. SDMs were trained using five widely applied and high‐performance algorithms—generalized linear models (GLM), generalized boosted regression models (GBM), maximum entropy (Maxent), RF and support vector machine (SVM); each repeated five times with bootstrapping replication using the ‘sdm’ function from the *sdm* R package. Model performance was assessed using true skill statistics (TSS), and only models with TSS ≥ 0.7 were retained. Ensemble models were constructed by weighted averaging these retained models according to their TSS performance scores.

To transform model predictions into binary presence/absence maps, we applied the maxSSS threshold (i.e., the value that maximizes the sum of sensitivity and specificity) across the models included in the ensemble (Liu et al. 2013). To reduce potential overprediction of suitable areas, we further constrained the binary maps using a buffered minimum convex polygon (MCP) around curated localities, following the method proposed by Kremen et al. (2008). As in earlier steps, the buffer scale ranged from 10 to 100 km depending on the size of the MCP (Brown et al. 2014). Finally, from each binary suitability map, we randomly sampled up to 5000 pixels (or fewer if the number of suitable pixels was lower) representing areas predicted as suitable habitat. Combined with the curated localities, these coordinates defined each species' climatic‐niche envelope, referred to hereafter as niche‐envelope localities (Datasets S3 and S4).

### 2.4 Correlation Coefficients Between ISD and Both Climatic and Geographic Centroids

To determine each species' climatic centroid, we extracted values from the seven selected bioclimatic variables at all niche‐envelope localities. We then applied a Principal Components Analysis (PCA) using the ‘prcomp’ function from the *stats* R package (R Core Team 2025), standardizing the input variables to zero mean and unit variance. The climatic centroid was defined as the mean position of each species along the first four principal component axes (PCs). On average, those four components captured 96.8% of the total climatic variance among niche‐envelope localities for reptiles (range: 89.5%–99.9% across 248 species) and 97.7% for amphibians (range: 92.5%–99.9% for 188 species). For the geographic centroid, we calculated the centroid of each species' IUCN range polygon using the ‘st_centroid’ function from the *sf* R package (Pebesma 2018; Pebesma and Bivand 2023). Alternative centroid definitions are provided in Appendix S2.

To explore whether ISD varied with spatial distance from the centroids, we computed (i) Euclidean distances between each niche‐envelope locality and the climatic centroid and (ii) Haversine distances between each niche‐envelope locality and the geographic centroid using the ‘distHaversine’ function from the *geosphere* R package (Hijmans 2024). ISD values for each locality were obtained from the interpolated nucleotide diversity surfaces. We then calculated species‐specific Spearman's *ρ* between ISD and each distance metric using the ‘cor.test’ function from the *stats* R package. To assess whether significantly negative correlations were more common than positive ones, we applied Pearson's Chi‐squared tests using the ‘chisq.test’ function from the *stats* R package.

### 2.5 Inference of the Main Predictors of Spearman's *ρ* Using RF

To model the species‐specific *ρ*—quantifying the relationship between ISD and distances to both climatic and geographic centroids, we assembled a comprehensive set of predictor variables (Datasets S5 and S6). These predictors describe: (i) the environmental conditions occupied by species; (ii) their ecological niches; (iii) spatial characteristics of species' ranges; (iv) morphological traits linked to dispersal potential; (v) genetic and demographic patterns; and (vi) data sampling effort (Table 2).

**TABLE 2:** Predictor variables used in the RF analyses explaining variation in Spearman's *ρ* values (i.e., correlation between intraspecific genetic diversity and distance to climatic or geographic centroids).

Category	Predictor	Description	Source
(i) Environmental	Bioclimatic variables (Bio1, Bio3, Bio5, Bio6, Bio12, Bio16, Bio17)	Climatic conditions	CHELSA
	Elevation	Altitudinal distribution	WorldClim
	Climate stability index	Historical climate variability	Herrando‐Moraira et al. (2022)
	Climate suitability	Probability of occurrence based on the SDMs	This study
(ii) Niche metrics	SNBT/SNBP	Species niche breadth for temperature and precipitation	This study
	Hypervolume	Multi‐dimensional climatic space	This study
	Marginality	How far a species' climatic niche is from the overall climatic centroid of all species	This study
(iii) Spatial	Latitude/longitude	Geographic positioning	This study
	Range size	Spatial extent of species distribution	IUCN
	Range eccentricity	Overall shape of the range	This study
	Realm	Wallace's zoogeographic realm	Holt et al. (2013)
(iv) Morphological	Maximum body mass, maximum length, maximum female length [reptiles]	Species relative size and proxy for dispersal	ReptTraits
	Body size [amphibians]	Species relative size and proxy for dispersal	AmphiBIO
(v) Genetic and demographic	Tajima's D	Indicator of demographic history or selection	This study
	ISD	Genetic diversity within species	This study
	Isolation‐by‐distance	Spatial genetic structure	This study
	Isolation‐by‐environment	Environmental genetic structure	This study
	mtGene	mtDNA gene analysed per species	GenBank
	Order, family	Phylogenetic classification	ReptileDatabase, AmphibiaWeb
(vi) Sampling	Sampling coverage	How thoroughly species' IUCN ranges were sampled	This study
	Relative representativeness	How well distributed were the sample points relative to a uniform distribution	This study
	Curated/niche localities	Number of curated localities and extracted from the SDMs	This study
	Interpolation sampling	Number of sequences included in the interpolations	This study
	Tajima's D/Mantel's test sampling	Number of sequences included in each test	This study

*Note:* Variables are grouped by category.

For the environmental predictors, we used the same seven bioclimatic variables employed in the SDMs at an ~1‐km resolution (see Section 2.3), supplemented with elevation data from Worldclim v.2.1 (Fick and Hijmans 2017). We also included the Climate Stability Index (CSI), a historical climate variability dataset spanning from the Pliocene to the present, obtained at an ~5‐km resolution (2.5 arc‐min; Herrando‐Moraira et al. 2022). All layers were harmonized to a common spatial resolution of ~2.5 km using bilinear interpolation using the ‘resample’ function from the *raster* R package (Hijmans 2025). Species‐specific values were extracted using the niche‐envelope localities using the ‘extract’ function from the *raster* R package and summarized as both mean and standard deviation (*σ*), capturing central tendency and variability for each predictor (Table 2). We also calculated the mean and *σ* of predicted climate suitability (probability of occurrence) extracted from the SDMs. We expected these predictors to influence ISD–centroid correlations because favourable, stable conditions and suitable climates should support larger, more persistent populations and therefore reinforce negative correlations with distance to climatic centroids, whereas species occupying variable or extreme environments may show weaker or even positive gradients.

Niche metrics were derived from SDM outputs. Niche breadth metrics were computed using annual extremes of temperature and precipitation, which are relevant to defining ecological tolerance and range limits (e.g., Liu et al. 2020; Moreira et al. 2024). Specifically, species niche breadth for temperature (SNBT) was defined as the range between the maximum temperature of warmest month (Bio5) and minimum temperature of coldest month (Bio6) values, while species niche breadth for precipitation (SNBP) was defined as the range between the precipitation of the wettest quarter (Bio16) and precipitation of driest quarter (Bio17) values, both across all niche‐envelope localities. To estimate niche hypervolume, we first conducted a PCA using the full set of species' niche‐envelope localities (separately for reptiles and amphibians), filtered this dataset per species, and then computed species‐specific hypervolumes using the first four PCs using the ‘hypervolume_gaussian’ function from the *hypervolume* R package (Blonder et al. 2015). This approach ensured comparability while reducing dimensionality. To assess niche marginality, we used the same PCA conducted on the full dataset (separately for reptiles and amphibians), calculated a grand centroid as the mean value of the first four PCs, filtered the PCA dataset per species, derived species‐specific centroids from the first four PCs, and then computed Euclidean distances between these centroids and the grand centroid. These predictors were expected to capture the role of ecological tolerance in shaping ISD–centroid relationships: species with broad climatic niches or large hypervolumes may exhibit weaker correlations due to greater tolerance of marginal conditions, while narrow‐niched species or those with high marginality may show steeper declines in ISD towards range or niche edges.

Spatial predictors included summary statistics (mean and *σ*) of latitude and longitude from the niche‐envelope localities. Range size (km^2^) was computed from IUCN polygons using the ‘st_area’ function from the *sf* R package. Eccentricity was calculated as the coefficient of variation in distances from the range centroid to 100 randomly sampled edge points, following Singhal et al. (2022). We also identified the dominant Wallace's Zoogeographic realm (Holt et al. 2013) based on the majority of occurrence records per species. We expected spatial attributes to mediate ISD–centroid patterns in several ways. Species occurring at lower latitudes generally harbour higher genetic diversity, which could increase the detectability of correlations. Larger geographic ranges were expected to provide greater statistical power to reveal such correlations, as they encompass broader spatial gradients. Conversely, highly eccentric ranges complicate the definition of a single geographic centroid, making distance‐based expectations less comparable across species. Realm identity may reflect historical and biogeographic contingencies that shape diversity patterns regionally.

For morphological predictors, we used body size as a proxy for dispersal capacity (Jenkins et al. 2007; Weil et al. 2023). Reptile traits (maximum body mass, length and female length) were sourced from ReptTraits (Oskyrko et al. 2024), while amphibian body size data were obtained from AmphiBIO (Oliveira et al. 2017). Missing values for body size were imputed using the ‘mice’ function from the *mice* R package (van Buuren and Groothuis‐Oudshoorn 2011), with 500 iterations incorporating family‐level taxonomy as a predictor. To improve accuracy, imputation was performed using the full ReptTraits and AmphiBIO datasets, covering ~12,000 reptile and ~6500 amphibian species, respectively. After imputation, we extracted the relevant body size values for our target species. Body size, as a proxy for dispersal ability, was expected to affect ISD–centroid correlations by modulating connectivity: larger‐bodied species may homogenize diversity across space, weakening gradients, while smaller‐bodied species may exhibit stronger declines in ISD towards the margins. Taxonomic assignments (order and family) were retrieved from the ReptileDatabase (Uetz et al. 2025) and AmphibiaWeb (AmphibiaWeb 2025).

Genetic and demographic predictors included Tajima's D, estimated using the ‘tajima.test’ function from the *pegas* R package. Tajima's D measures departures from the neutral equilibrium model of molecular evolution: values close to zero indicate neutrality, while significantly negative or positive values reflect demographic processes such as population expansion, bottlenecks, or balancing speciation (Tajima 1989). We retained both the test statistic and associated *p*‐value, identifying whether species deviated from equilibrium expectations. Two additional predictors assessed population structure: isolation‐by‐distance (IBD) and isolation‐by‐environment (IBE), following Pelletier and Carstens (2018). IBD was estimated using the ‘mantel’ function from the *vegan* R package (Oksanen et al. 2025) with 1000 permutations and Pearson correlation. IBE was calculated using the ‘mantel.partial’ function, controlling for geographic distance while testing the effect of environmental distance on genetic divergence. For both tests, we retained the correlation coefficients and corresponding *p*‐values. We also included the mean and *σ* of ISD (interpolated values at the niche‐envelope localities) and recorded the mtDNA gene used for each species. These variables were expected to capture demographic history and structure: negative Tajima's D values may indicate expansions that weaken gradients, while positive values (bottlenecks or balancing selection) could accentuate them. Similarly, strong IBD or IBE may reinforce spatial structuring of diversity, leading to stronger correlations with centroid distance. Lastly, we incorporated taxonomic predictors, namely the order and family for each species, to account for the non‐independence of closely related taxa.

We also included sampling‐related predictors. Sampling coverage was assessed as the proportion of the species range covered by niche‐envelope localities within a 100 km buffer. To evaluate how representative our samples were relative to species ranges, we used the ‘urap.proportion.held’ function from the *raptr* R package (Hanson et al. 2018). This metric compares observed sample distributions to a uniform distribution across the species' range. For expected representativeness, we generated evenly distributed sample points using k‐means clustering on raster centroids, selecting cluster centroids as reference points. Voronoi polygons were computed using the *dismo* R package (Hijmans et al. 2024), and the same representativeness metric was calculated for these simulated points. We then computed the ratio between observed and expected representativeness as a final metric. Sampling‐related predictors were included to evaluate how data quality and coverage might influence the detectability of ISD–centroid relationship. Better and more representative sampling should provide greater statistical power to detect correlations. Additional metrics of sampling effort included: (i) number of sequences used in the interpolation (see Section 2.2); (ii) number of curated and niche localities (see Section 2.3); (iii) number of sequences in the Tajima's D test; and (iv) number of sequences in the Mantel's test.

To investigate which variables best explain *ρ*, we implemented a machine learning approach using RF (Breiman 2001). This method constructs an ensemble of decision trees (i.e., a ‘forest’), each of which makes predictions based on a subset of the data and predictors. The importance of each predictor is quantified by evaluating how much the model's prediction error increases when that variable is excluded (Liaw and Wiener 2002). We conducted a RF regression analysis using the *caret* R package (Kuhn 2008). Prior to model training, we calculated pairwise Pearson correlation coefficients (*r*) among all predictor variables (53 for reptiles and 51 for amphibians) using the ‘cor’ function from the *stats* R package. To reduce multicollinearity, we excluded predictors with *r* > 0.80. The dataset was then split into a training set (90%) and a testing set (10%).

We first ran an initial RF with 1000 trees to obtain a ranked list of predictors based on their importance, using the ‘importance’ function from the *randomForest* R package (Liaw and Wiener 2002). From this preliminary model, we retained the top 20 most important predictors for further analysis. We then built a final RF model, also using 1000 trees, and performed five‐fold cross‐validation to optimize the analysis. This procedure was repeated across 50 different random seeds to ensure stability of the importance ranking, and the variable importance values were averaged across runs to produce a consensus ranking. Model performance was evaluated using the root mean squared error (RMSE). To further explore the relationship between *ρ* and the top predictors, we generated scatterplots depicting each top predictor's association with Spearman's *ρ* values.

## 3 Results

Data cleaning and filtering yielded a final dataset of 436 species distributed across six major global regions, consisting of 248 reptiles and 188 amphibians. All retained species met predefined thresholds for sequence alignment and spatial coverage, although sampling effort varied among species. On average, reptile species were represented by ~75 mtDNA sequences (range: 10–537) across ~35 unique georeferenced localities (range: 5–233), whereas amphibians were represented by ~99 sequences (range: 10–1350) across ~28 localities (range: 5–523). Representation varied across regions (Table 1). For reptiles, coverage was highest in the Iberian Peninsula, where nearly 69% of all native species were included, followed by Northwestern Africa (~40%) and the Arabian Peninsula (~33%). Representation was lower in Madagascar and the Australian Tropics (~12% each) and especially in the Brazilian Atlantic Forest (~10%). Amphibians showed a similar pattern: sampling was highest in the Iberian Peninsula (~69%) and Northwestern Africa (~46%), moderate in the Arabian Peninsula (~36%), and lower in the Australian Tropics (~19%), the Brazilian Atlantic Forest (~15%) and Madagascar (~12%).

**FIGURE 3:**
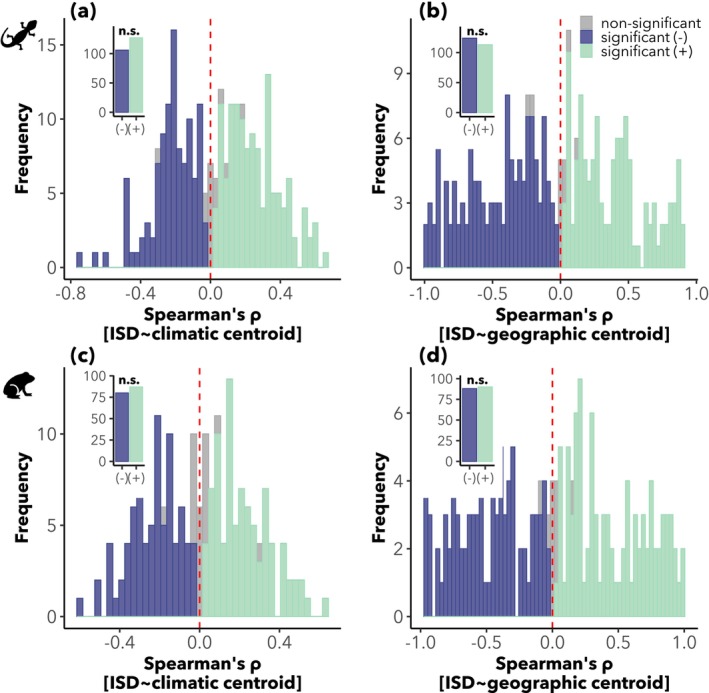
Distribution of Spearman's *ρ* values describing the correlation between intraspecific genetic diversity (ISD) and distance to species centroids for reptiles and amphibians. Panels a and b show the correlation with distance to the climatic and geographic centroids, respectively, for reptiles (*n* = 248 species); panels c and d show the same for amphibians (*n* = 188 species). In each histogram, insets within each panel show the number of species with significantly positive and negative *ρ* (*p* < 0.05). The label n.s. above each inset indicates that the difference in counts is not statistically significant (Pearson's Chi‐squared test). Overall, results highlight the lack of consistent directional trends in ISD–centroid correlations across both taxonomic groups and distance metrics.

The Spearman's *ρ*, which describes the relationship between ISD and the distance to either climatic or geographic centroids, exhibited considerable variation across species (Datasets S7 and S8). Patterns were highly heterogeneous among herptile species, with no clear directional trend in ISD–centroid relationships. While many correlations were statistically significant, the distribution of *ρ* values was roughly centred around zero. There were no detectable biases towards more positive or more negative correlations (Figure 3).

**FIGURE 4:**
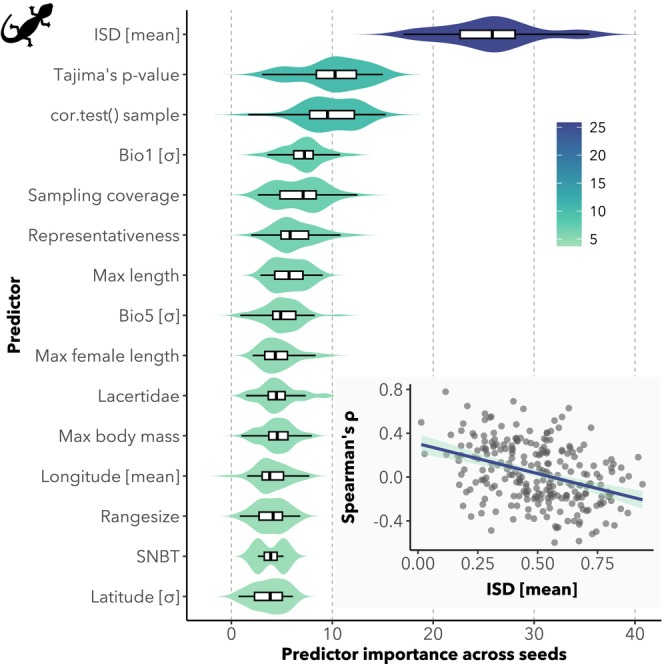
Variable importance scores for the top 15 predictors from the RF model explaining variation in Spearman's *ρ* values describing the correlation between intraspecific genetic diversity (ISD) and distance to the climatic centroid in reptiles (*n* = 248 species). Violin plots show the distribution of importance scores across repeated model runs (*n* = 50), with internal boxplots indicating medians and interquartile ranges. The colour palette reflects the relative ranking of variable importance, with darker tones indicating higher importance. *σ*, standard deviation; Bio1, mean annual air temperature; Bio5, mean daily maximum air temperature of the warmest month; ISD, intraspecific genetic diversity; SNBT, species niche breadth. Bottom‐right panel: Scatterplot illustrates the relationship between species' mean ISD and their corresponding Spearman's *ρ* values. Grey points represent individual species; the fitted linear regression line (dark purple) and 95% confidence interval (light blue) reveal a tendency for species with higher mean ISD to show more negative ISD–centroid correlations.

We trained a total of 200 RF regression models using a set of 41 predictor variables for reptiles and 40 for amphibians, encompassing ecological, spatial and methodological traits. RF models focused on explaining the relationship between ISD and distance to climatic centroids in reptiles accounted for an average of 11.9% ± 2.3% of the variance in *ρ*. From these models, variable importance scores highlighted mean ISD as an important predictor of *ρ* (Figure 4; Dataset S9). A negative relationship was found between mean ISD and *ρ* values (Figure 4), indicating that species with higher mean ISD tend to show stronger negative correlations—suggesting that ISD decreases more sharply towards niche edges in these species. Nonetheless, the predictive contribution of mean ISD was limited and only relevant in the context of climatic centroid models for reptiles (Dataset S10). In all other cases—including geographic centroids for reptiles, and both climatic and geographic centroids for amphibians—the models performed poorly, with average *R* ^2^ values ranging from −3.8% to −1.5%. These findings were robust to several complementary sensitivity tests. Specifically, (i) incorporating multiple mtDNA genes per species, rather than retaining only the most representative locus, did not systematically shift inferred ISD–distance to centroid relationships (Figure S2); (ii) alternative definitions of climatic and geographic centroids (i.e., using a minimum volume ellipsoid instead of PCA‐derived climatic centroids, and a MCP instead of the IUCN polygon centroid for geographic centroids) did not alter these relationships (Figure S3); (iii) restricting analyses to curated localities rather than the full set of niche‐envelope localities yielded consistent results (Figure S4); and (iv) excluding species with relative representativeness scores below 50 produced qualitatively similar outcomes (Figures S5–S7; Appendix S3; Datasets S11 and S12).

## 4 Discussion

ISD plays a pivotal role in shaping species' adaptive potential and long‐term persistence. A prevailing assumption in biogeography and conservation is that ISD tends to decline towards range margins, a hypothesis known as the central‐marginal paradigm (Eckert et al. 2008). However, using a comprehensive dataset of 436 reptile and amphibian species across six regions, we were unable to detect any consistent spatial pattern in ISD. Instead, correlations between ISD and distance to geographic or climatic centroids are highly variable in both magnitude and direction, defying any single overarching rule. This result mirrors findings from other recent comparative studies and adds further weight to the growing body of evidence challenging the universality of the central‐marginal hypothesis (Lira‐Noriega and Manthey 2014; Singhal et al. 2022). Interestingly, while our results reveal little consistency in how ISD varies with distance to centroids, recent global analyses of demographic responses suggest that niche structure can be a strong predictor of species' sensitivity to environmental pressures. In amphibians, for example, Cordier et al. (2025) showed that niche breadth and the position of populations relative to the climatic centroid strongly influence vulnerability to habitat alteration, with marginal populations generally less resilient. This contrast underscores that genetic diversity patterns may be shaped by processes not fully captured by niche structure alone. While some species in our dataset exhibited higher ISD near the geographic (e.g., *Ctenotus spaldingi*, *Bokermannohyla saxicola* ) or climatic centroid (e.g., *Tropiocolotes algericus*, *Hoplobatrachus tigerinus* )—consistent with those theoretical expectations—others showed the opposite trend, with greater genetic diversity at the range periphery (e.g., *Echis coloratus* , *Phyllomedusa bahiana* ) or towards climatic margins (e.g., *Madascincus polleni*, *Hyla meridionalis* ). These contrasting patterns likely reflect differences in demographic history, dispersal capacity and habitat configuration, such as reduced connectivity or stronger drift towards range margins in some species, versus long‐term persistence in peripheral refugia or environmentally heterogeneous regions in other species. A substantive number of species showed no significant trend at all. This mixture of outcomes was reflected in the near‐symmetrical distribution of species‐specific Spearman's *ρ* values centred around zero, with no significant differences in the frequency of positive vs. negative values.

Very little of the observed variation in ISD–centroid correlation strength can be explained by the ecological, spatial, or methodological predictors utilized here. The RF models, which incorporated more than 40 predictors related to climate, ecology, geography, morphology and demography, all performed poorly such that the best model explained just under 12% of the variance in *ρ*. For all other taxonomic groups and centroid definitions, model performance was negligible (*R* ^2^ < 2.5%). These results echo earlier interpretations (Lira‐Noriega and Manthey 2014; Singhal et al. 2022) that ISD patterns are largely idiosyncratic and lineage‐specific. Among the predictors examined, mean ISD was consistently the most influential in the best‐performing model. Notably, we observed a negative relationship between mean ISD and the correlation strength (*ρ*)—reptiles with higher overall genetic diversity tended to show more strongly negative correlations with distance to climatic centroids. Despite considerable scatter (Figure 4, bottom‐right panel), this negative association may reflect a stronger central‐peak structure in ISD among genetically diverse species (i.e., high mean ISD). However, this relationship was restricted to a subset of the data and offered little predictive power when applied across taxa, limiting its general utility.

Our findings underscore the difficulty of generalizing ISD patterns and challenge the assumption that an unstudied species will follow the same spatial trends observed in related or co‐occurring taxa. Each species' genetic structure likely reflects a unique combination of evolutionary history, demographic events and environmental interactions that may not be easily captured by broad‐scale predictors. Empirical studies have shown that the spatial scale at which genetic structure emerges can vary markedly among species, often reflecting dispersal capacity and landscape configuration. For instance, fine‐scale phylogeographic analyses of low‐dispersal taxa have revealed deep and spatially complex genetic structure over only a few kilometres, even when sampling spans the entire known range of the species (*Xerocrassa montserratensis*; Català et al. 2021). Similarly, multi‐site studies in tropical trees show that the strength and scale of fine‐scale genetic structure can differ markedly among species and across sites within the same species, reflecting interactions between dispersal traits and local environmental heterogeneity rather than uniform, range‐wide processes (Smith et al. 2018). The poor performance of our models therefore raises an important question: what environmental filters or demographic processes are shaping ISD patterns that we are failing to capture? Potential factors include fine‐scale habitat heterogeneity, historical climate refugia that may have influenced demographic history, past range expansions or contractions of subpopulations, and unmeasured ecological interactions. Moreover, key demographic drivers of ISD—such as local population density, migration rates and dispersal barriers—are extremely difficult to summarize at the scale of this study. These metrics are often missing or unevenly reported across species and regions, yet they can exert strong influences on spatial genetic patterns. Additionally, although we deliberately selected regions spanning distinct biomes and biogeographic contexts, regional inclusion was constrained by data availability and taxonomic expertise. As with most macrogenetic studies, these choices limit inference for other regions (e.g., North America), underscoring the need for future extensions as comparable datasets become available. Until such data can be incorporated more systematically, attempts to generalize ISD structure will remain limited.

An important consideration in interpreting our results concerns the biological meaning and spatial scale of the analytical units used to estimate ISD. In this study, ISD was calculated from spatially explicit interpolations of nucleotide diversity derived from georeferenced sequences, rather than from discrete, independently defined populations. As a result, the spatial ‘bins’ used here should not be interpreted as demographic populations, but rather as continuous representations of genetic diversity across species' ranges. Leigh et al. (2021) recommend pooling genomic data at the population level in macrogenetic studies to preserve biologically meaningful units, and we agree that this represents an ideal framework where population boundaries can be robustly defined. However, defining populations at a global scale presents substantial challenges, particularly for taxa with limited sampling, uneven geographic coverage, or poorly resolved population structure. In such cases, population delineation may introduce additional subjectivity and inconsistency across species, potentially biasing comparative analyses. Our approach therefore aimed to minimize sampling‐density effects while enabling standardized, range‐wide comparisons across hundreds of species.

Our findings were robust to several sensitivity tests explicitly designed to evaluate whether methodological choices influenced the inferred ISD–distance to centroid relationships. Specifically, re‐analyses using alternative definitions of geographic and climatic centroids, incorporating multiple mtDNA genes per species, distinguishing between genetically georeferenced localities and climatically predicted niche‐envelope localities, and excluding species with relative representativeness scores below 50 all yielded similar results. These analyses reinforce the conclusion that the lack of generality is not driven by methodological artefacts, but instead reflects underlying biological complexity. In tandem with similar studies, we show that the central‐marginal hypothesis does not hold uniformly across taxa (Lira‐Noriega and Manthey 2014; Singhal et al. 2022). It is therefore inappropriate to assume that an unstudied species will exhibit spatial genetic patterns similar to those observed in related taxa or within the same region. Conservation strategies that prioritize range centres without empirical validation risk overlooking key populations that harbour unique or high levels of genetic diversity.

An important caveat of our work is the reliance on mtDNA as the primary genetic marker. mtDNA is widely used in comparative studies because of its broad availability across taxa, yet it represents only a small, maternally inherited portion of the genome, which does not recombine and may therefore provide an incomplete picture of genome‐wide diversity (Galtier et al. 2009; Larkin et al. 2023). As a result, mtDNA diversity does not necessarily mirror nuclear or genome‐wide diversity, nor does it consistently capture the adaptive potential of populations. Differences in effective population size or inheritance mode, for example, mean that mtDNA and nuclear markers can respond differently to demographic history and spatial or environmental gradients. Indeed, recent work has shown that mtDNA and nuclear genetic diversity can exhibit contrasting demographic patterns, even at broad spatial scales (Clark and Pinsky 2024). These considerations caution against using mtDNA alone to guide conservation decisions (Schmidt and Garroway 2021). However, our objective here was not to quantify the absolute magnitude of within‐population genetic diversity or to treat mtDNA as a direct proxy for genome‐wide variation. Rather, we tested whether broad‐scale correlations between ISD and distance to climatic or geographic centroids emerge consistently across many species when assessed using standardized and widely available markers. If general spatial rules governing genetic diversity were pervasive, they might be expected to manifest even in mtDNA data, which often capture historical demography and large‐scale population structure. The absence of consistent ISD–centroid relationships across species therefore suggests that such general rules are weak or highly contingent, rather than simply obscured by marker choice. With the increasing accessibility of genome‐wide datasets, future studies will be able to revisit these questions using nuclear markers and explicitly test whether the patterns we observe hold at the genomic level. In addition, the amount and type of mtDNA data available varied among species. Some taxa were represented by a single mtDNA locus with limited geographic coverage, whereas others had data available for multiple mtDNA genes and a greater number of sequences. This heterogeneity reflects the structure of publicly available databases and may contribute additional noise to cross‐species comparisons; the original references for all sequences used in this study are compiled in Datasets S13 and S14. To mitigate this, we standardized our main analyses by retaining the most representative mtDNA gene per species, and conducted complementary sensitivity analyses integrating multiple loci where available, as well as excluding species with low relative representativeness scores. The persistence of highly variable ISD–distance to centroid relationships across these alternative analyses suggests that the absence of consistent spatial patterns is not solely driven by differences in mtDNA gene representation among species.

An additional consideration concerns the uneven genetic and spatial sampling available across species. While species with large geographic ranges were sometimes represented by relatively few samples, the number of sequences alone does not fully capture how well genetic diversity is represented. Spatial distribution and representativeness of samples across the range are equally critical. For this reason, we explicitly quantified sampling coverage and representativeness and incorporated these metrics as predictors in our RF analyses. Nevertheless, true species‐wide genetic diversity is unknown a priori, and a given level of sampling may adequately capture diversity in some species but not in others, depending on dispersal, demographic history and population structure. These limitations are inherent to large‐scale macrogenetic studies relying on publicly available data and should be considered when interpreting range‐wide patterns of ISD. Consistent with this, we find weak or no relationships between species' range size and either representativeness or the number of sequences used for interpolation (Figure S8), indicating that broad geographic ranges do not necessarily imply better genetic coverage.

In summary, our study highlights the absence of universal spatial rules governing ISD in herptiles, despite consistent methodology and broad‐scale data integration. This calls for a shift in conceptualization for macrogenetics research: from seeking simple generalizations to embracing species‐specific, data‐informed approaches that acknowledge the evolutionary and ecological intricacies underlying genetic diversity patterns in nature.

## Author Contributions

M.O.M. conceived the study, led to data acquisition, curation and analysis, developed the methodological framework, interpreted the results, and wrote and revised the manuscript. M.J.P. contributed to data extraction and curation, methodological development, data analysis and manuscript review. A.V.L. assisted with data extraction and manuscript review. A.C.C. and B.C.C. contributed to methodological decisions, interpretation of results and critical manuscript revisions. S.B.C. was responsible for the study conception, data acquisition and curation, methodological design, interpretation of results, supervision and manuscript reviewing.

## Funding

This work was funded by Fundação para a Ciência e Tecnologia, I.P, via national funds, under the scope of project PLACES—‘PLAnning for the Conservation of life on Earth in the light of evolution and Sustainability’ (https://doi.org/10.54499/2022.08134.PTDC). S.B.C. was supported by Fundação para a Ciência e Tecnologia through the individual scientific employment program contract (https://doi.org/10.54499/2022.04598.CEECIND/CP1730/CT0006). A.V.L. was supported by Xunta de Galicia (ED481B‐2025/025).

## Conflicts of Interest

The authors declare no conflicts of interest.

## Supporting information


**Appendix S1:** Average nucleotide diversity across mtDNA loci.
**Appendix S2:** Alternative distance metrics for climatic and geographic centroids.
**Appendix S3:** RF analyses after filtering species with low relative representativeness scores.
**Figure S1:** Schematic overview of the pipeline used to estimate intraspecific genetic diversity (ISD) across species' ranges.
**Figure S2:** Distribution of Spearman's *ρ* values describing the correlation between intraspecific genetic diversity (ISD) and distance to species centroids for reptiles and amphibians. Panels a and b show the correlation with distance to the climatic and geographic centroids, respectively, for reptiles (*n* = 248 species); panels c and d show the same for amphibians (*n* = 188 species). Unlike the main analysis, ISD values were calculated using multiple mtDNA genes per species whenever more than one gene was available. Insets within each panel show the number of species with significantly positive and negative *ρ* (*p* < 0.05). The label n.s. above each inset indicates that the difference in counts is not statistically significant (Pearson's Chi‐squared test).
**Figure S3:** Distribution of Spearman's *ρ* values describing the correlation between intraspecific genetic diversity (ISD) and distance to species centroids for reptiles and amphibians. Panels a and b show the correlation with distance to the climatic and geographic centroids, respectively, for reptiles (*n* = 248 species); panels c and d show the same for amphibians (*n* = 188 species). Alternative methods were used to calculate distance metrics: a minimum volume ellipsoid (MVE) was used for the climatic centroid, and a minimum convex polygon (MCP) was used for the geographic centroid. Insets within each panel show the number of species with significantly positive and negative *ρ* (*p* < 0.05). The label n.s. above each inset indicates that the difference in counts is not statistically significant (Pearson's Chi‐squared test).
**Figure S4:** Distribution of Spearman's *ρ* values describing the correlation between intraspecific genetic diversity (ISD) and distance to species centroids for reptiles and amphibians. Panels a and b show the correlation with distance to the climatic and geographic centroids, respectively, for reptiles (*n* = 248 species); panels c and d show the same for amphibians (*n* = 188 species). This analysis is based on curated localities only, rather than niche‐envelope localities. Insets within each panel show the number of species with significantly positive and negative *ρ* (*p* < 0.05). The label n.s. above each inset indicates that the difference in counts is not statistically significant (Pearson's Chi‐squared test).
**Figure S5:** Distribution of relative representativeness values for reptiles (*n* = 248 species) and amphibians (*n* = 188 species). Violin plots show the distribution of relative representativeness scores across groups, with internal boxplots indicating medians and interquartile ranges. On average, reptile species have representativeness scores of ~77 (Q1: 72; median: 88; Q3: 95), whereas amphibians have representativeness scores of ~74 (Q1: 67; median: 85; Q3: 94). The dashed red line represents the threshold of 50, where species on the left have relative representativeness scores below this value.
**Figure S6:** Distribution of Spearman's *ρ* values describing the correlation between intraspecific genetic diversity (ISD) and distance to species centroids for reptiles and amphibians. Panels a and b show the correlation with distance to the climatic and geographic centroids, respectively, for reptiles (*n* = 211 species); panels c and d show the same for amphibians (*n* = 157 species). This analysis is based on species with relative representativeness scores above or equal to 50. Insets within each panel show the number of species with significantly positive and negative *ρ* (*p* < 0.05). The label n.s. above each inset indicates that the difference in counts is not statistically significant (Pearson's Chi‐squared test).
**Figure S7:** Variable importance scores for the top 15 predictors from the RF model explaining variation in Spearman's *ρ* values describing the correlation between intraspecific genetic diversity (ISD) and distance to the climatic centroid in reptiles (*n* = 211 species). This analysis is based on species with relative representativeness scores above or equal to 50. Violin plots show the distribution of importance scores across repeated model runs (*n* = 50), with internal boxplots indicating medians and interquartile ranges. The colour palette reflects the relative ranking of variable importance, with darker tones indicating higher importance. Bottom‐right panel: scatterplot illustrates the relationship between species' mean ISD and their corresponding Spearman's *ρ* values. Grey points represent individual species; the fitted linear regression line (dark purple) and 95% confidence interval (light blue) reveal a tendency for species with higher mean ISD to show more negative ISD–centroid correlations.
**Figure S8:** Relationships between species' geographic range size and sampling metrics for reptiles and amphibians. Top‐left panel shows the relationship between IUCN range size (km^2^) and relative representativeness for reptiles, while the top‐right panel shows range size versus the number of sequences used in the spatial interpolations for reptiles. Bottom‐left and bottom‐right panels show the corresponding relationships for amphibians, plotting range size against relative representativeness and interpolation sample size, respectively. Points represent individual species. Together, these plots illustrate that species with larger geographic ranges are not necessarily better represented genetically, with relationships generally weak or absent across groups.

## Data Availability

The datasets and R code used for the analyses are available on Zenodo (https://doi.org/10.5281/zenodo.17314952).
