# Supplementary material for: Spatial Patterns of Intraspecific Genetic Diversity Follow no General Rule Across Climatic and Geographic Gradients
Source: Mol Ecol. 2026 Mar 24;35(6):e70321. doi: 10.1111/mec.70321 (PMC13010785; doi:10.1111/mec.70321)

**Supplementary Information for**

Spatial patterns of intraspecific genetic diversity follow no general rule across climatic and geographic gradients

**Contents (in this file)**

Appendices S1–S3

Figures S1–S8

**Other materials (in separate files) are available on Zenodo:**

Datasets S1–S14

R scripts

Zenodo link: https://doi.org/10.5281/zenodo.17314952

**APPENDIX S1 |** Average nucleotide diversity across mtDNA loci.

To derive a species-level estimate of average intraspecific genetic diversity (ISD) across mtDNA loci, we combined locus-specific interpolations of nucleotide diversity using a spatially weighted approach. For each species with more than one mtDNA locus available, we first delineated the convex hull of its IUCN range to define boundary cells and calculated the maximum pairwise geographic distance among these points. This value represented the maximum spatial extent of the species’ distribution and was used to normalize distance-based weights across loci. For each mtDNA gene with at least five unique georeferenced localities, we calculated the great-circle distance between every grid cell within the species’ range and all available sampling points for that gene. The mean distance to the five nearest sampling locations was computed for each cell, normalized by the maximum boundary distance, and then converted into an inverse distance weight (1 – normalized distance), such that grid cells nearer to observed localities received higher weights. These weights were stored as separate raster layers corresponding to each gene.

Gene-specific rasters of nucleotide diversity (previously interpolated using spatial splines; see section 2.2 of the main text) were then multiplied by their respective inverse distance weight rasters, yielding a set of weighted diversity surfaces. The weighted mean ISD was calculated cell by cell across all loci as the ratio between the sum of weighted nucleotide diversity values and the sum of their corresponding weights. This procedure integrates information across multiple loci while accounting for spatial heterogeneity in sampling density, ensuring that loci with broader and denser spatial coverage contribute proportionally more to the final estimate. The resulting weighted mean raster thus represents a spatially explicit, multi-locus measure of ISD, summarizing overall genetic diversity patterns across each species’ geographic range.

**APPENDIX S2 |** Alternative distance metrics for climatic and geographic centroids.

To derive an alternative estimate of each species’ climatic centroid, we first extracted the values of the seven bioclimatic variables (i.e., Bio1, Bio3, Bio5, Bio6, Bio12, Bio16, Bio17) at all niche-envelope localities. We randomly partitioned these data into a training set (70%) and a testing set (30%), and then removed variables exhibiting a variance inflation factor (VIF) greater than 10 to reduce multicollinearity. Using the training set, we built niche models with two, three, or four of the most informative climatic predictors and evaluated model fit using the ‘ellipsoid_selection’ function from the *ntbox* R package (Osorio‐Olvera et al., 2020), following the approach of Singhal et al. (2022). This procedure defined the species’ niche as a minimum volume ellipsoid (MVE), whose center was taken as the climatic centroid. Mahalanobis distances from each niche-envelope locality to this climatic centroid were then calculated, using the ‘mahalanobis’ function from the *stats* R package (R Core Team, 2025).

For a revised estimate of geographic centroid, we constructed a minimum convex polygon (MCP) around each species’ niche-envelope localities using the ‘st_convex_hull’ function from the *sf* R package (Pebesma, 2018; Pebesma & Bivand, 2023). We then computed the Haversine distance between each locality and the MCP-derived centroid using the ‘distHaversine’ function from the *geosphere* R package (Hijmans, 2024).

**APPENDIX S3 |** RF analyses after filtering species with low relative representativeness scores.

To evaluate the sensitivity of our results to uneven spatial sampling, we conducted an additional set of RF analyses after excluding species with low sampling representativeness. Specifically, we removed all species with a relative representativeness (RelREP) value below 50, a threshold chosen to retain species whose genetic sampling more adequately reflects their modeled geographic range. The distribution of relative representativeness values for reptiles and amphibians, prior to filtering, is illustrated in Figure S5.

Following this filtering step, 37 reptile species and 31 amphibian species were excluded from the dataset. The RF models were then re-run using the same predictor variables, tuning parameters, and analytical framework as in the main analyses.

The qualitative outcomes of these filtered analyses were consistent with those obtained using the full dataset. The Spearman’s ρ, which describes the relationship between ISD and the distance to either climatic or geographic centroids, exhibited considerable variation across species. Patterns were highly heterogeneous among herptile species, with no clear directional trend in ISD-centroid relationships. There were no detectable biases toward more positive or more negative correlations (Figure S6). RF models focused on explaining the relationship between ISD and distance to climatic centroids in reptiles accounted for an average of 14.4% ± 3.1% of the variance in ρ. From these models, variable importance scores highlighted mean ISD as an important predictor of ρ (Figure S7; Dataset S11). A negative relationship was found between mean ISD and ρ values (Figure S7), indicating that species with higher mean ISD tend to show stronger negative correlations. Nonetheless, the predictive contribution of mean ISD was limited and only relevant in the context of climatic centroid models for reptiles (Dataset S12). In all other cases – including geographic centroids for reptiles, and both climatic and geographic centroids for amphibians – the models performed poorly, with R² values ranging from -11.6 to 3.8. This indicates that our core conclusions are robust to the exclusion of species with lower spatial sampling representativeness.

**REFERENCES**

Hijmans, R. J. (2024). geosphere: spherical trigonometry. *R Package Version 1.5-20, <https://CRAN.R-Project.Org/Package=geosphere>*.

Osorio‐Olvera, L., Lira‐Noriega, A., Soberón, J., Peterson, A. T., Falconi, M., Contreras‐Díaz, R. G., Martínez‐Meyer, E., Barve, V., & Barve, N. (2020). ntbox: an R package with graphical user interface for modelling and evaluating multidimensional ecological niches. *Methods in Ecology and Evolution*, *11*(10), 1199–1206. https://doi.org/10.1111/2041-210X.13452

Pebesma, E. (2018). Simple features for R: standardized support for spatial vector data. *The R Journal*, *10*(1), 439–446. https://doi.org/10.32614/RJ-2018-009

Pebesma, E., & Bivand, R. (2023). *Spatial data science: with applications in R*. Chapman and Hall/CRC. https://doi.org/10.1201/9780429459016

R Core Team. (2025). R: A language and environment for statistical computing. *R Foundation for Statistical Computing, Vienna, Austria. <https://Www.R-Project.Org/>*.

Singhal, S., Wrath, J., & Rabosky, D. L. (2022). Genetic variability and the ecology of geographic range: a test of the central‐marginal hypothesis in Australian scincid lizards. *Molecular Ecology*, *31*(16), 4242–4253. https://doi.org/10.1111/mec.16589

To ensure appropriate attribution of primary data sources, we have compiled the original references for all sequences used in this study in Datasets S13 and S14.**FIGURE S1 |** Schematic overview of the pipeline used to estimate intraspecific genetic diversity (ISD) across species' ranges.


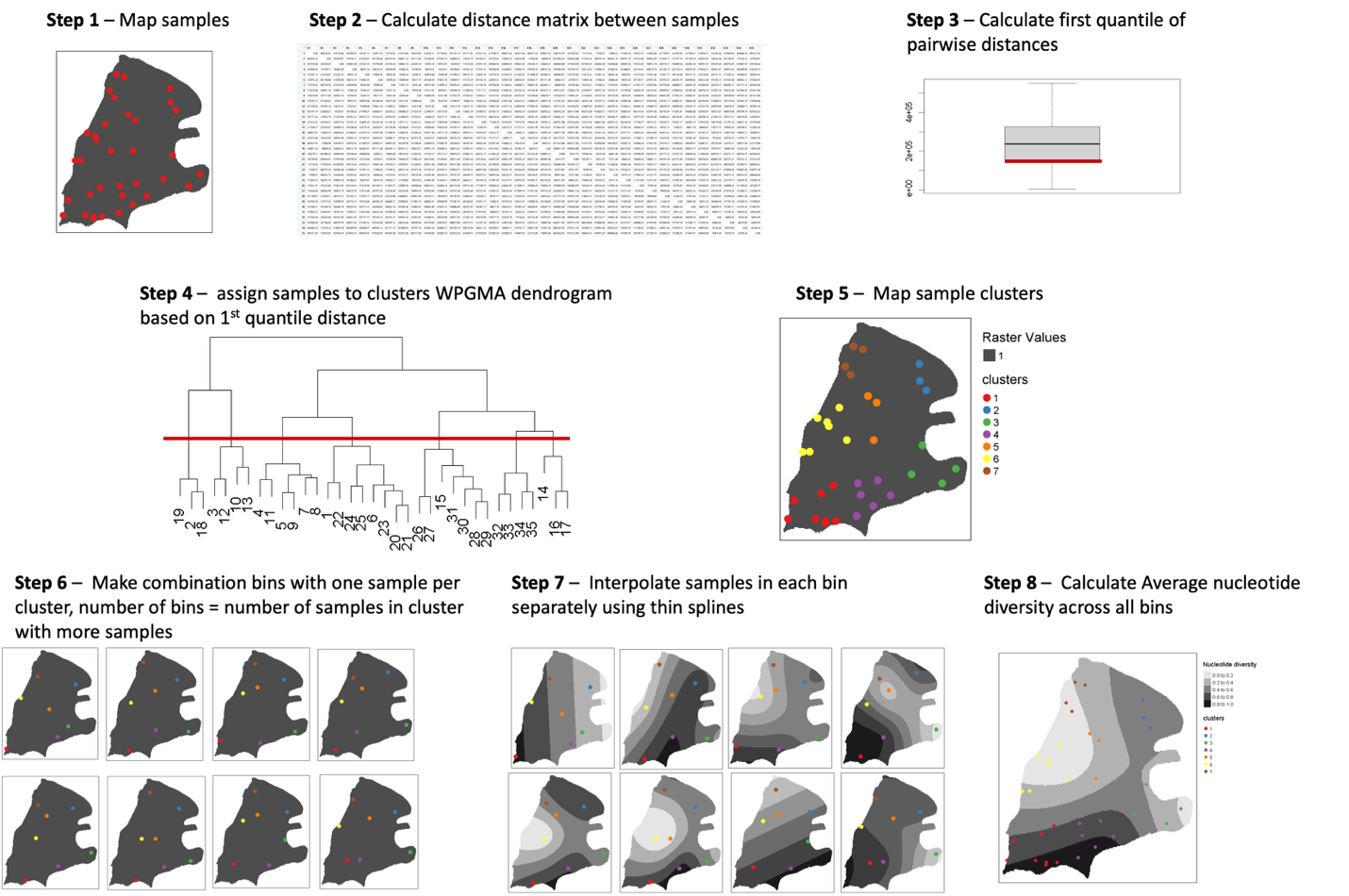


**FIGURE S2 |** Distribution of Spearman’s ρ values describing the correlation between intraspecific genetic diversity (ISD) and distance to species centroids for reptiles and amphibians. Panels **a** and **b** show the correlation with distance to the climatic and geographic centroids, respectively, for reptiles (n = 248 species); panels **c** and **d** show the same for amphibians (n = 188 species). Unlike the main analysis, ISD values were calculated using multiple mtDNA genes per species whenever more than one gene was available. Insets within each panel show the number of species with significantly positive and negative ρ (p < 0.05). The label n.s. above each inset indicates that the difference in counts is not statistically significant (Pearson’s Chi-squared test).


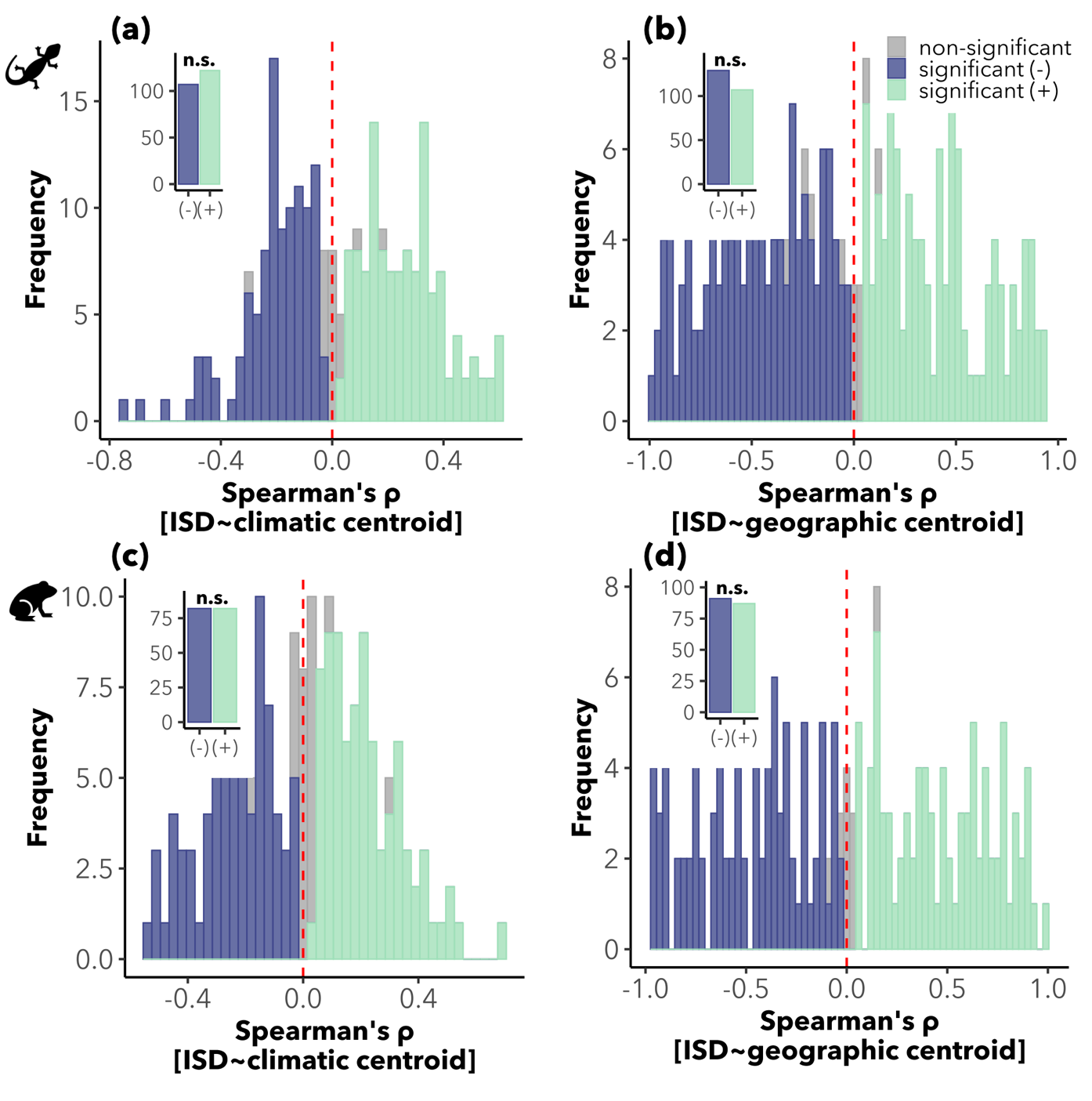


**FIGURE S3 |** Distribution of Spearman’s ρ values describing the correlation between intraspecific genetic diversity (ISD) and distance to species centroids for reptiles and amphibians. Panels **a** and **b** show the correlation with distance to the climatic and geographic centroids, respectively, for reptiles (n = 248 species); panels **c** and **d** show the same for amphibians (n = 188 species). Alternative methods were used to calculate distance metrics: a minimum volume ellipsoid (MVE) was used for the climatic centroid, and a minimum convex polygon (MCP) was used for the geographic centroid. Insets within each panel show the number of species with significantly positive and negative ρ (p < 0.05). The label n.s. above each inset indicates that the difference in counts is not statistically significant (Pearson’s Chi-squared test).

**
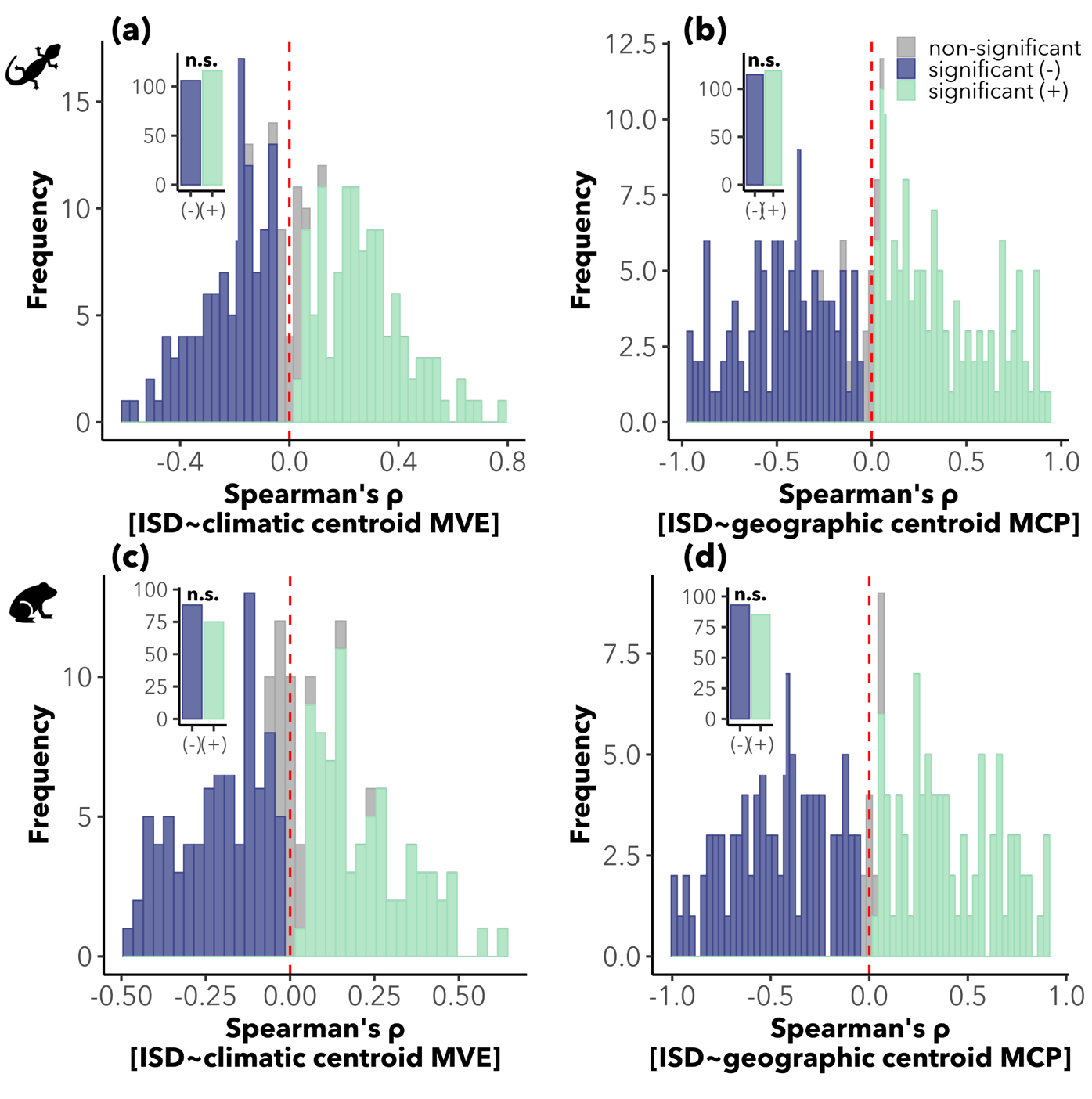
**

**FIGURE S4 |** Distribution of Spearman’s ρ values describing the correlation between intraspecific genetic diversity (ISD) and distance to species centroids for reptiles and amphibians. Panels **a** and **b** show the correlation with distance to the climatic and geographic centroids, respectively, for reptiles (n = 248 species); panels **c** and **d** show the same for amphibians (n = 188 species). This analysis is based on curated localities only, rather than niche-envelope localities. Insets within each panel show the number of species with significantly positive and negative ρ (p < 0.05). The label n.s. above each inset indicates that the difference in counts is not statistically significant (Pearson’s Chi-squared test).

**
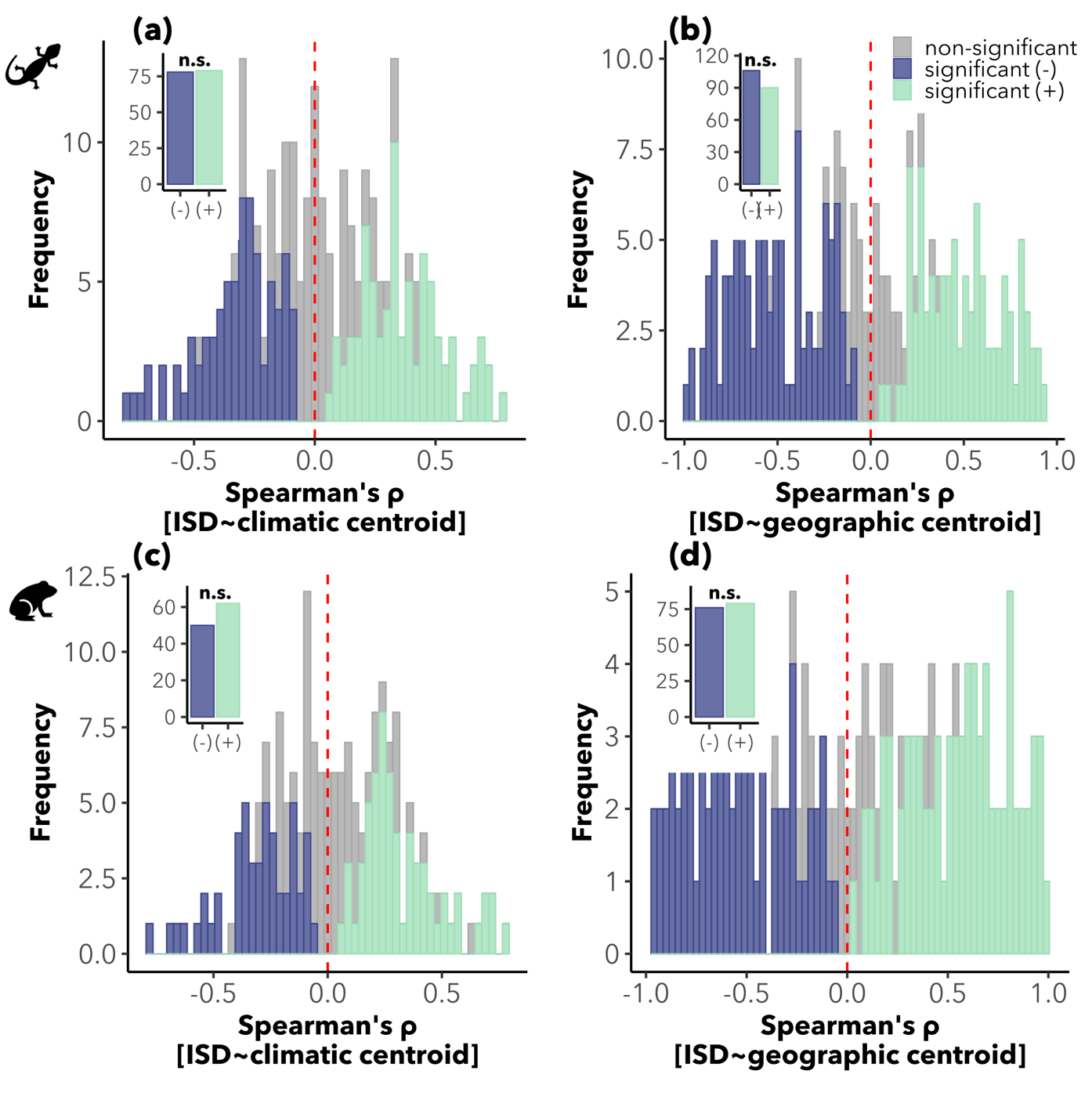
**

**FIGURE S5 |** Distribution of relative representativeness values for reptiles (n = 248 species) and amphibians (n = 188 species). Violin plots show the distribution of relative representativeness scores across groups, with internal boxplots indicating medians and interquartile ranges. On average, reptile species have representativeness scores of ~77 (Q1: 72; median: 88; Q3: 95), whereas amphibians have representativeness scores of ~74 (Q1: 67; median: 85; Q3: 94). The dashed red line represents the threshold of 50, where species on the left have relative representativeness scores below this value.

**
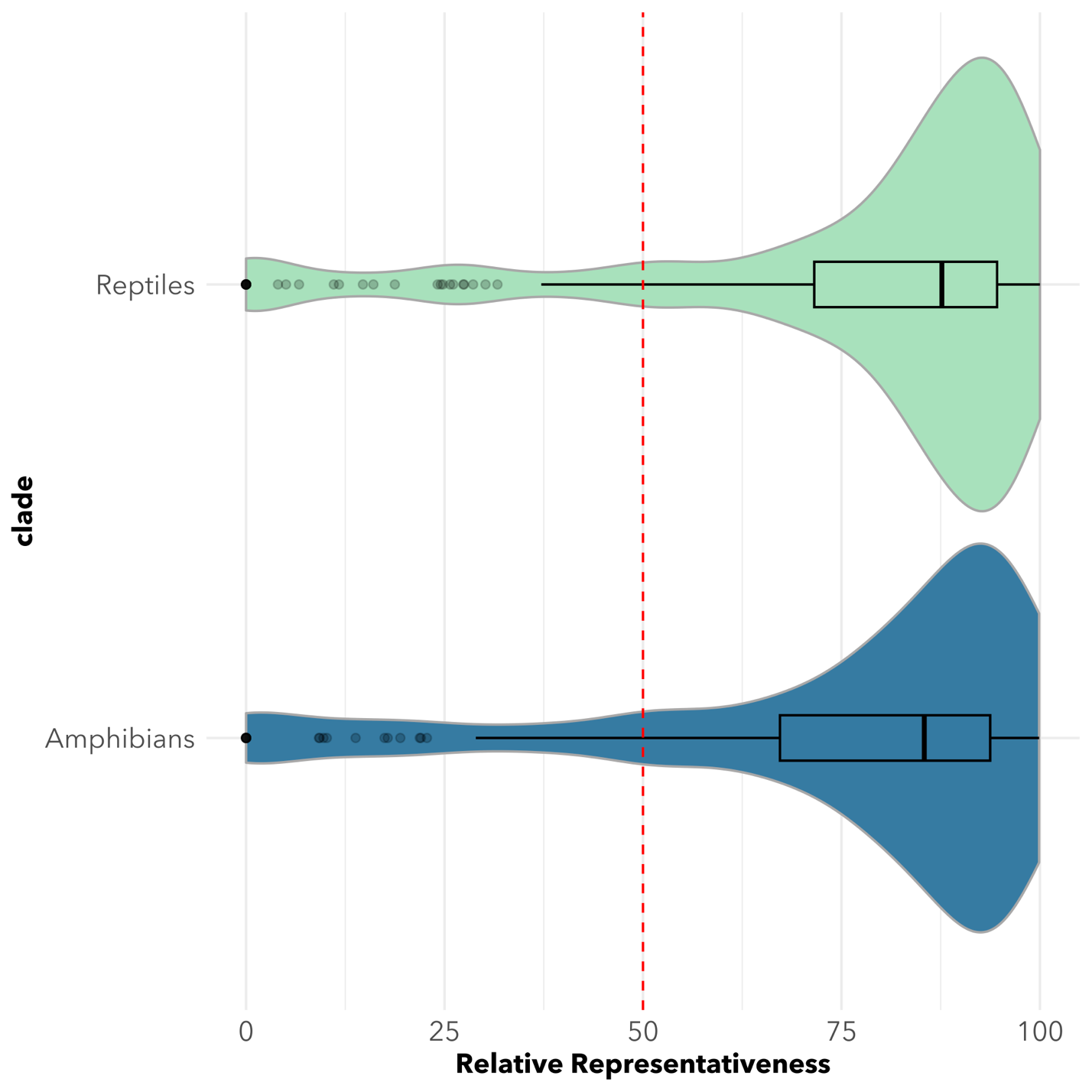
**

**FIGURE S6 |** Distribution of Spearman’s ρ values describing the correlation between intraspecific genetic diversity (ISD) and distance to species centroids for reptiles and amphibians. Panels **a** and **b** show the correlation with distance to the climatic and geographic centroids, respectively, for reptiles (n = 211 species); panels **c** and **d** show the same for amphibians (n = 157 species). This analysis is based on species with relative representativeness scores above or equal to 50. Insets within each panel show the number of species with significantly positive and negative ρ (p < 0.05). The label n.s. above each inset indicates that the difference in counts is not statistically significant (Pearson’s Chi-squared test).


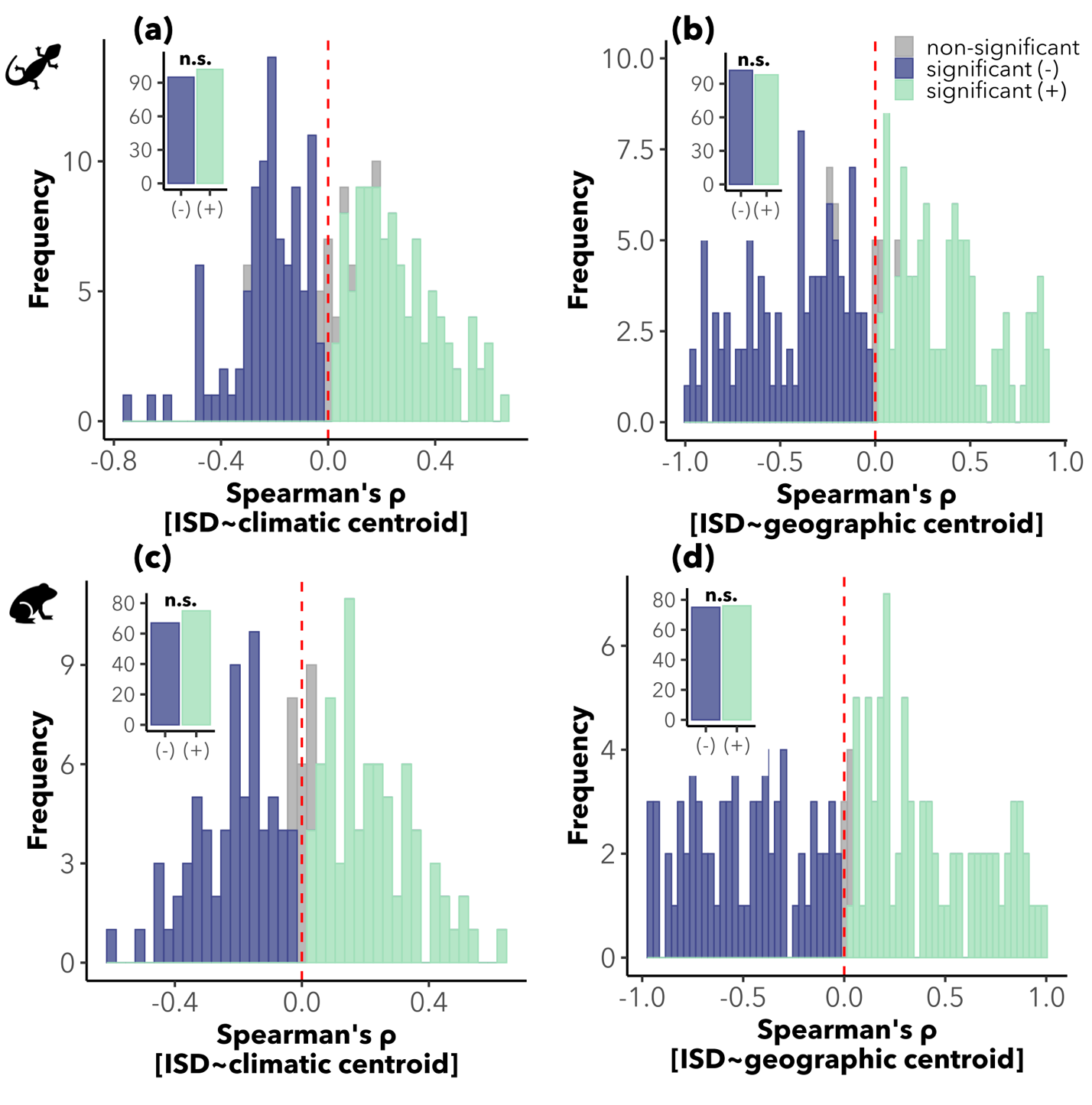


**FIGURE S7 |** Variable importance scores for the top 15 predictors from the RF model explaining variation in Spearman’s ρ values describing the correlation between intraspecific genetic diversity (ISD) and distance to the climatic centroid in reptiles (n = 211 species). This analysis is based on species with relative representativeness scores above or equal to 50. Violin plots show the distribution of importance scores across repeated model runs (n = 50), with internal boxplots indicating medians and interquartile ranges. The color palette reflects the relative ranking of variable importance, with darker tones indicating higher importance. Bottom-right panel: scatterplot illustrates the relationship between species’ mean ISD and their corresponding Spearman’s ρ values. Grey points represent individual species; the fitted linear regression line (dark purple) and 95% confidence interval (light blue) reveal a tendency for species with higher mean ISD to show more negative ISD-centroid correlations.

**
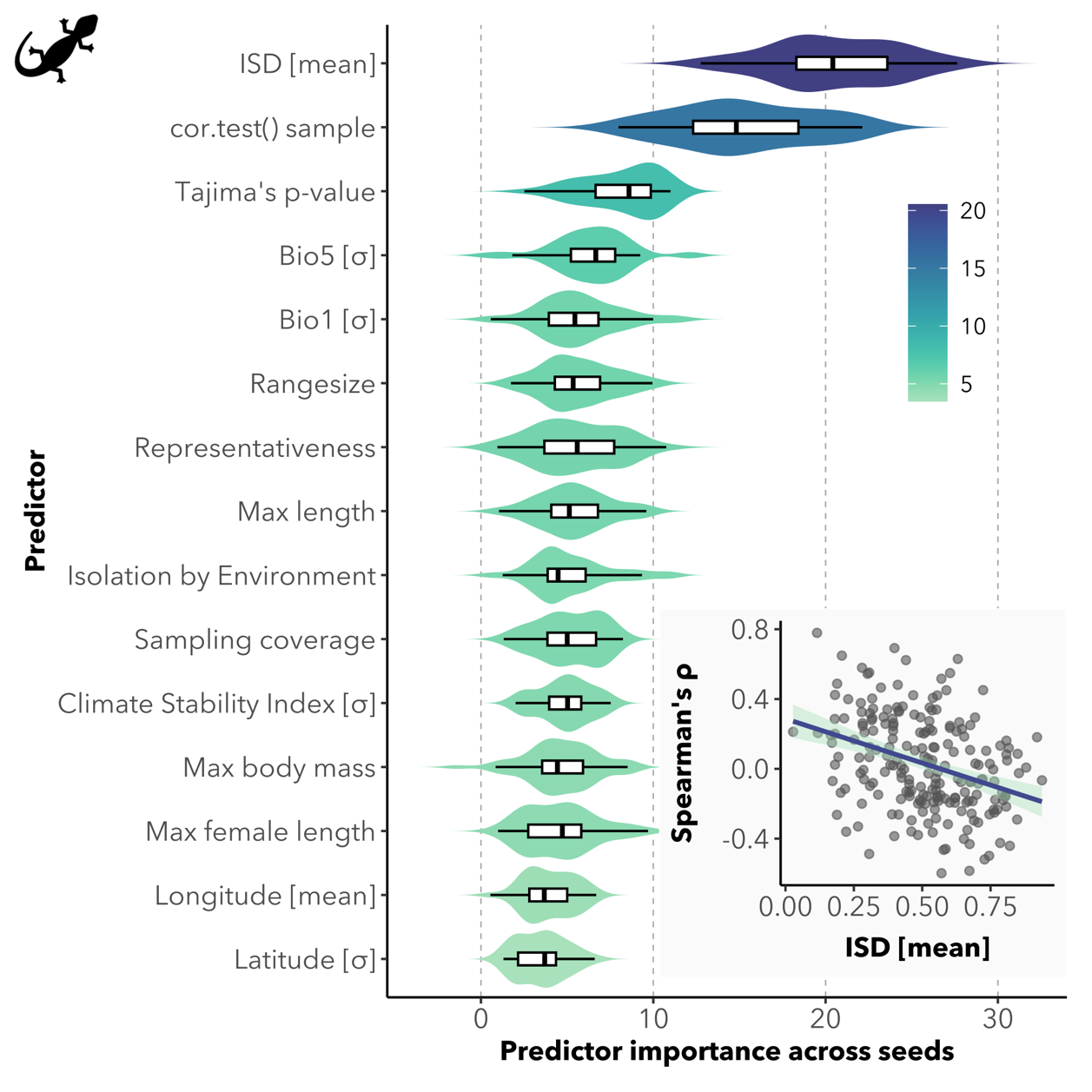
**

**FIGURE S8 |** Relationships between species’ geographic range size and sampling metrics for reptiles and amphibians. Top-left panel shows the relationship between IUCN range size (km²) and relative representativeness for reptiles, while the top-right panel shows range size versus the number of sequences used in the spatial interpolations for reptiles. Bottom-left and bottom-right panels show the corresponding relationships for amphibians, plotting range size against relative representativeness and interpolation sample size, respectively. Points represent individual species. Together, these plots illustrate that species with larger geographic ranges are not necessarily better represented genetically, with relationships generally weak or absent across groups.


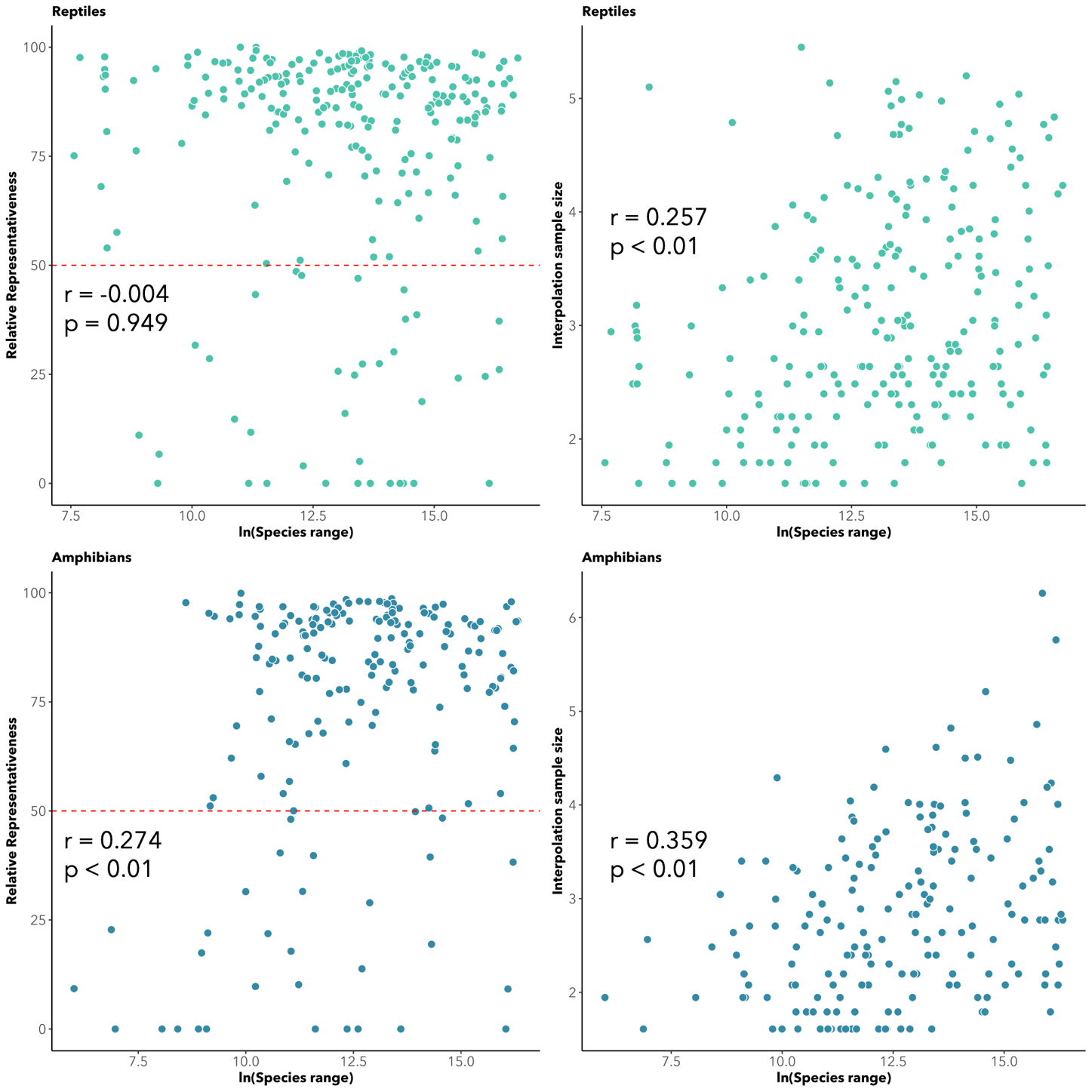

Supplement: Supplementary file 1 — Appendix S1: Average nucleotide diversity across mtDNA loci. Appendix S2: Alternative distance metrics for climatic and geographic centroids. Appendix S3: RF analyses after filtering species with low relative representativeness scores. Figure S1: Schematic overview of the pipeline used to estimate intraspecific genetic diversity (ISD) across species' ranges. Figure S2: Distribution of Spearman's ρ values describing the correlation between intraspecific genetic diversity (ISD) and distance to species centroids for reptiles and amphibians. Panels a and b show the correlation with distance to the climatic and geographic centroids, respectively, for reptiles (n = 248 species); panels c and d show the same for amphibians (n = 188 species). Unlike the main analysis, ISD values were calculated using multiple mtDNA genes per species whenever more than one gene was available. Insets within each panel show the number of species with significantly positive and negative ρ (p < 0.05). The label n.s. above each inset indicates that the difference in counts is not statistically significant (Pearson's Chi‐squared test). Figure S3: Distribution of Spearman's ρ values describing the correlation between intraspecific genetic diversity (ISD) and distance to species centroids for reptiles and amphibians. Panels a and b show the correlation with distance to the climatic and geographic centroids, respectively, for reptiles (n = 248 species); panels c and d show the same for amphibians (n = 188 species). Alternative methods were used to calculate distance metrics: a minimum volume ellipsoid (MVE) was used for the climatic centroid, and a minimum convex polygon (MCP) was used for the geographic centroid. Insets within each panel show the number of species with significantly positive and negative ρ (p < 0.05). The label n.s. above each inset indicates that the difference in counts is not statistically significant (Pearson's Chi‐squared test). Figure S4: Distribution of Spearman's ρ values de [file MEC-35-e70321-s001.docx]
